# Newsprint coverage of smoking in cars carrying children: a case study of public and scientific opinion driving the policy debate

**DOI:** 10.1186/1471-2458-14-1116

**Published:** 2014-10-29

**Authors:** Shona Hilton, Karen Wood, Josh Bain, Chris Patterson, Sheila Duffy, Sean Semple

**Affiliations:** MRC/CSO Social and Public Health Sciences Unit, University of Glasgow, Top Floor, 200 Renfield Street, Glasgow, G2 3QB UK; Action on Smoking & Health (Scotland), 8 Frederick Street, Edinburgh, EH2 2HB UK; Scottish Centre for Indoor Air, Division of Applied Health Sciences, University of Aberdeen, AB25 2ZD Aberdeen, UK

## Abstract

**Background:**

Media content has been shown to influence public understandings of second-hand smoke. Since 2007 there has been legislation prohibiting smoking in all enclosed public places throughout the United Kingdom (UK). In the intervening period, interest has grown in considering other policy interventions to further reduce the harms of second-hand smoke exposure. This study offers the first investigation into how the UK newsprint media are framing the current policy debate about the need for smoke-free laws to protect children from the harms of second-hand smoke exposure whilst in vehicles.

**Methods:**

Qualitative content analysis was conducted on relevant articles from six UK and three Scottish national newspapers. Articles published between 1^st^ January 2004 and 16^th^ February 2014 were identified using the electronic database Nexis UK. A total of 116 articles were eligible for detailed coding and analysis that focused on the harms of second-hand smoke exposure to children in vehicles.

**Results:**

Comparing the period of 2004–2007 and 2008–2014 there has been an approximately ten-fold increase in the number of articles reporting on the harms to children of second-hand smoke exposure in vehicles. Legislative action to prohibit smoking in vehicles carrying children was largely reported as necessary, enforceable and presented as having public support. It was commonly reported that whilst people were aware of the general harms associated with second-hand smoke, drivers were not sufficiently aware of how harmful smoking around children in the confined space of the vehicle could be.

**Conclusions:**

The increased news reporting on the harms of second-hand smoke exposure to children in vehicles and recent policy debates indicate that scientific and public interest in this issue has grown over the past decade. Further, advocacy efforts might draw greater attention to the success of public-space smoke-free legislation which has promoted a change in attitudes, behaviours and social norms. Efforts might also specifically highlight the particular issue of children’s developmental vulnerability to second-hand smoke exposure, the dangers posed by smoking in confined spaces such as vehicles, and the appropriate measures that should be taken to reduce the risk of harm.

## Background

Since 2007 there has been legislation prohibiting smoking in all enclosed public places throughout the UK [1], with Scotland being the first to implement the law in 2006 [2]. In the intervening period interest has grown in considering other policy interventions to further reduce the harmful effects of second-hand smoke (SHS) exposure to children. This interest largely stems from fears that following the implementation of the legislation, smoking would be displaced to the home environment. However, evidence suggests that this did not occur and that a by-product of the legislation is that there has been an increase in the number of smoke-free homes [3, 4].

One explanation for the widespread acceptability of the legislation is that it may have reflected a growing awareness about the harms of SHS exposure and marked a shift in attitudes towards the need for legislation to protect vulnerable groups, such as children [5]. This may have arisen from the intense media reporting and high profile public health campaigns about the harms of SHS that preceded and accompanied the introduction of the legislation. Similar high levels of compliance following positive media reporting have occurred in other countries after the introduction of similar smoke-free laws [6].

Kitzinger [7] notes that the level of media attention correlates with the degree of salience these issues have for the public and that public concern. Policy attention rises and falls in response to shifts in media coverage rather than with any changes in the actual size of the problem in the real world. Thus, the more news coverage an issue receives, the more important the issue may be perceived to be. With indoor public spaces no longer a major source of SHS exposure, the micro-environments where exposure continues include the private spaces of the vehicle and home. There appears to be little appetite for legislation on restricting smoking in the home [8] but the situation with respect to smoking in vehicles is more open to debate with health professionals, charities and politicians arguing the case for restrictions since 2007 [9–11].

Scotland's recently published ‘Tobacco Control Strategy’ includes a commitment to reducing people’s exposure to SHS and to setting a target to reduce children’s exposure [12]. One commitment in the strategy is the need for a social marketing campaign to highlight the dangers of SHS to children in confined spaces and there is some evidence to suggest that such a campaign might find public support. For example, a recent British Lung Foundation (BLF) survey of 8–15 year olds found that 86% of the children who took part supported legislation to prohibit smoking in vehicles carrying children [13–15]. Further, since the private space of the vehicle is already subject to legislation, ranging from restrictions on smoking in work vehicles, mobile phone usage, laws on the use of seat belts and child-baby carriers; further legislation in this area might be seen as palatable to the public.

Looking wider afield, some states and provinces in the United States, Canada and Australia have already introduced legislation prohibiting smoking in vehicles carrying children [16]. In the UK the British Medical Association has called for further action on smoking in vehicles [17] and at a devolved level the Welsh assembly has recently announced that legislation banning smoking in vehicles carrying children will be introduced, and the Northern Irish assembly has called for increased awareness raising about the issue, with the prospect of legislative changes should the education approach not bear fruit [18]. In Scotland, on 28^th^ May 2013, MSP Jim Hume proposed a draft ‘Smoking (Children in Vehicles) (Scotland) Bill’ to prohibit smoking in private vehicles. On 30^th^ January 2014, the final proposal was lodged at the Scottish Parliament, achieving the necessary cross-party support from MSPs (at least 18 signatures) to proceed on the first day of proposal. Additionally, in England on the 10^th^ February 2014, the House of Commons passed an amendment to the Children and Families Bill, empowering ministers to introduce legislation preventing smoking in vehicles carrying children [19]. As noted by Seale [20] empirical research on the role of the media in the development of health policy is an underdeveloped area. Yet having a more nuanced understanding of how the debate is being framed by the media will offer new insights into the role the news media play in propagating ideas about the acceptability of further smoke-free laws to protect children. This study aims to examine how the newsprint media have reported the debate about protecting children from SHS in cars over the past 10 years with the aim of providing public health advocates with useful insights for future communication strategies.

## Method

We selected nine newspapers (six published across the UK and three published specifically for a Scottish readership) with their corresponding Sunday editions. This created a total sample of 18 newspapers. Of these eight were ‘serious’ newspapers (formerly known as ‘broadsheets’), four were ‘middle-market’ tabloid newspapers and six were ‘tabloid’ newspapers. This typology has been used in other newspaper analyses to represent a range of readership profiles diverse in terms of age, social class, and political ideology [21]. A time frame of 1^st^ Jan 2004 to 31^st^ Dec 2013 was selected to allow a baseline measure of news reporting on SHS prior to the enactment of the first UK smoke-free legislation in Scotland in 2006. This timeframe was then extended to 16^th^ February 2014 to take account of articles published the week following the amendment to the Children and Families Bill on the 10^th^ February 2014. Articles were identified using the electronic database Nexis UK. The search terms used were (where ‘!’ indicates a wildcard): *“smok! OR tobacco OR cig! OR second hand smok! OR passive smok!” AND “babies OR baby OR child! OR kid! OR infant! OR early years OR toddler! OR tot! OR parent! OR mum! OR dad! OR car! OR vehicle!”.*

The search yielded 422 news articles. All these articles were read by two researchers using inclusion and exclusion criteria. Articles were excluded if: the content did not relate to issues reporting on SHS in vehicles and its effects on children; they were published in Irish (Eire) editions of the newspapers; they were duplicate articles, letters, advice, TV guides, sport, weather, obituaries and review pages. Following the filtering process, a total of 116 articles were deemed eligible for detailed coding and analysis. These news articles were re-read and thematically coded using a qualitative software program NVivo 10 to organise data. Written summaries of these thematic categories were developed and cross-checked by three researchers (SH, KW, JB). To identify patterns across the data the constant comparative method [22, 23] was adopted. What emerged from the articles were themes around the dominant ideas and arguments about the rationale, feasibility to developing smoke-free vehicle laws in the UK, and arguments presented in opposition.

## Results

Over the past decade, 116 news articles reported on SHS in vehicles and its effects on children in these newspapers. Of these articles 40.5% (n = 47) were published in ‘serious’ newspapers, 31.9% (n = 37) in ‘mid-market’, and 27.6% (n = 32) in ‘tabloid’ newspapers. In the period leading up to the introduction of the Scottish, Northern Irish and Welsh smoke-free public places legislative changes (with exceptions in Wales and Northern Ireland), and the English smoke-free work places legislative changes (between 2004 and 2007) only seven articles (6.0% of the total identified) were published relating to SHS in vehicles and its effects on children. However from 1^st^ January 2008 to 16^th^ February 2014, 109 articles (94.0% of the total identified) were published, with the highest annual rate of publication occurring in 2011 (n = 32). Comparing the ‘baseline’ period 2004–2007 (generally prior to implementation of smoke-free laws in public places) with the period 2008–2014 suggests a ten-fold increase in reporting on the topic of SHS in vehicles and its effects on children.

From our analysis of the 116 news articles three dominant themes emerged: 111 articles mentioned the problem of SHS vehicle exposure, 91 articles mentioned arguments reporting on the feasibility of smoke-free vehicle laws as a policy solution to the problem, and 65 articles mentioned the counter-arguments.

### Key arguments presented to highlight the problem of SHS vehicle exposure to children

1. *SHS exposure is a major health risk to children*
  Almost all of the articles reported that SHS was harmful to the health of children. A wide range of respiratory conditions, illnesses and diseases were attributed to the effects of SHS, with some articles highlighting the ongoing risks to health in later life such as the risk of developing cancer. It was reported that: “children were at particular risk of damage from SHS due to their faster breathing rates and less developed immune systems” (The Scotsman, 16^th^ Oct 2012). Further, there was a tendency to highlight the differences between adults and children to demonstrate the developmental vulnerability of children.
2. *There is a dangerously high level of SHS exposure in confined spaces like vehicles*
  Vehicles were described as one of the main places of exposure to SHS remaining for children following the smoke-free laws. Various figures and statistics were reported throughout the news articles as evidence of the scale of the problem and to highlight how many children were regularly being exposed to SHS while in vehicles. It was common for articles to emphasise the issue of vehicles being a ‘confined space’ and that this posed a greater risk because of the high concentrations of harmful particles which could exceed air-quality standards. Children were described as ‘confined’ ‘trapped’ and ‘legally exposed’ to breathe in harmful pollutants. To further highlight the point a few articles compared levels of SHS in vehicles in the UK with: “industrial smog in cities such as Beijing or Moscow…” (The Herald, 7^th^ Sept 2011), and with smoke levels found in bars pre-legislation (The Express, 18^th^ Jun 2009).
3. *Drivers are unaware that opening the window is not enough*
  Linked to the above argument was the reporting that opening a window was an insufficient response to these “poisonous particles” (Journalist, The Scotsman, 20^th^ Jan 2011) and that it was not suffice to protect their children from the harms (Daily Record, 28^th^ May 2013). This led to reporting that people were well aware of the harms associated with SHS, but that people were often unaware of how harmful smoking in vehicles could be to children breathing in that smoke.
4. *Adults that smoke in vehicles carrying children are irresponsible, child needs protected from them*
  Another key theme to emerge as an argument for smoke-free vehicle laws was the issue that there is a duty to protect children from harms of “thoughtless”, “seriously bad” (Journalist, The Sunday Herald, 21^st^ Sept 2009), “selfish” (Journalist, Daily Star, 20^th^ Jan 2011) parents, and that only people “… with half a brain would poison a car full of kids with fag smoke” (Journalist, The Express, 25^th^ Mar 2010). These parents were described as “knowing what they’re doing. And that’s why legislation is probably, albeit unfortunately, necessary” (The Sunday Herald, 21^st^ Sept 2009).

### Arguments reporting on the feasibility of smoke-free vehicle laws as a policy solution to the problem

1. *Legislative action is necessary*
  The current situation for children was described as being ‘unfair’ and as ‘requiring intervention’ in several articles. BLF and ASH Scotland spokespersons often were quoted as stating that a law to prevent smoking in vehicles would be justified on the basis of children’s health ‘alone’. It was suggested that: “As a society, creating such a measure is a powerful statement of intent about our commitment to the health of our children” (Daily Star, 7^th^ Oct 2010). Some policy advocates went further arguing that children have: “the right not to be harmed” (Jim Hume, Liberal Democrat Party Member of the Scottish Parliament, The Scotsman, 29^th^ May 2013) and to be protected (Alex Cunningham – Labour Party Member of Parliament, Daily Record, 23^rd^ Jun 2011).
2. *Legislative action is enforceable*
  It was pointed out that the vehicle is actually a ‘semi-public space’ (Daily Mail, 16^th^ Sept 2009). One editorial in The Scotsman stated: “Critics, of course, do not question an extension of the ban to cars as such, but argue it would be unenforceable. But it is no more so than compulsory seatbelts or a ban on dangerous driving. The law reaches into cars already. And the vast majority would accept the legitimacy of a smoking ban” (Editorial, The Scotsman, 24^th^ Mar 2010). Many articles reported claims that publicity and education campaigns were not enough to change people’s behaviour, suggesting that nudging people to change their behaviours had been shown, “to fail time and again” (BMA, The Daily Telegraph, 16^th^ Nov 2011). It also emerged from the articles that several other countries had already introduced similar legislation and that it had good public support and had been enforceable.
3. *Legislative action changes attitudes*
  A number of opinion polls were also reported across the news articles suggesting that the majority of people would support a legislative action and that it would likely lead to a further changes in people’s attitudes towards the social acceptability of smoking around children. One article described legislation as “a benchmark of decency and declaring through law that something is unacceptable”. Noting the same goes for all other areas of public life where something that used to be tolerated has been ruled to have no part in modern life. (Journalist, The Guardian, 31^st^ May 2013). In this sense legislative action was also presented as building on past legislation and on public support for existing smoke-free legislation (BMA, The Daily Telegraph, 16^th^ Nov 2011).

### Presenting the counter-arguments

1. *A lack of evidence on the harms of SHS exposure to children in vehicles*
  There were some opposing voices challenging the assertion that SHS is harmful to children and questioning the strength of evidence on SHS. The tobacco industry funded lobby group, ‘Forest’, offered quotes throughout news articles over the decade describing the evidence as “weak” (Daily Record, 16^th^ November 2011). It was also claimed that the evidence for the dangers of SHS was “based on junk statistics” (Libertarian Alliance, Daily Mail, 17^th^ Nov 2011) and to infer that the risks from SHS exposure were deliberately being exaggerated.
2. *The wrong focus for legislative action*
  It was suggested that other sources of environmental pollution were far more dangerous to people’s health: “the greatest environmental health risk comes not from cigarette smoke but pollution caused by power stations and car exhausts” (Journalist, The Sunday Herald, 28^th^ Mar 2009). It was also suggested that there are more dangerous threats to children’s health, listing: “poor diets, no sport, illiteracy, homelessness, emotional abuse, female circumcision, parental absenteeism” as examples (Journalist, The Observer, 20^th^ Nov 2011). Critics argued that instead of legislation, information and education campaigns would be more successful in stopping parents from smoking in vehicles, “education, not coercion is the solution” (Daily Mail, 16^th^ Sept 2009). Legislation was described as: “heavy-handed” (Simon Clark, spokesperson for Forest, The Daily Telegraph, 17^th^ Jun 2009) and an over-reaction to the scale of the problem: “using a jackhammer to crack a nut” (Journalist, The Observer, 20^th^ Nov 2011).
3. *Unenforceable legislation*
  Across the news articles critics suggested that the legislation would be “difficult” to enforce (The Express, 16^th^ Jul 2011) with lobbyists describing it as “almost impossible” (Forest, Daily Mail, 30^th^ Mar 2007). Questions were raised around who would enforce the legislation given the cuts to police budgets, and it was argued that the legislation would be another: “example of the diversion of police away from their essential business of stopping real crime” (MP, Daily Mail, 17^th^ Nov 2011). Reference was also made to the potential confusion arising from one country enacting legislation while the neighbouring country did not, leading drivers unintentionally to break the law.
4. *Erosion of smokers’ rights*
  Commonly cited in articles that reported opposition to the legislation was claims about smokers’ rights being “under threat” (Forest, Daily Mail, 30^th^ Mar 2007), “eroded” (Forest, Daily Mail, 1^st^ Feb 2010) and “breached” (Journalist, Daily Star, 7^th^ Oct 2010). It was argued that this legislation in vehicles would go “beyond what is acceptable in a free society” (Forest, Daily Mail, 24^th^ Mar 2010) and it was presented that: “…we all have the right to make certain choices free from state interference.” (Editorial, The Scotsman, 16^th^ Oct 2012). Another article described smokers as: “the most harassed, demonised and bullied community in Britain today” (Journalist, The Mirror, 25^th^ Mar 2010). It was also argued that it was only a small step towards further restrictions on where people were allowed to smoke in their homes and if laws in vehicles were successful it would be a “triumph for the nanny state” (MP, Daily Mail, 1th Nov 2011).

## Discussion

This study offers some of the first insights into how the UK newsprint media are framing the current policy debate about the need for smoke-free vehicle laws to protect children from the harms of SHS exposure. The key findings from our analysis are that the increased news reporting on the harms of SHS exposure to children in vehicles and recent policy debates indicate that scientific and public interest in this issue has grown over the past decade. Further, legislative action to prohibit smoking in vehicles carrying children was largely reported as necessary, enforceable and presented as having public support, and it was commonly reported that whilst people were aware of the general harms associated with SHS, drivers were not sufficiently aware of how harmful smoking around children in the confined space of the vehicle could be.

The tobacco industry has a formidable record of resisting legislation and of developing new marketing strategies, including strategies of trying to keep smoking in public view against a backdrop of it becoming an increasingly de-normalised pubic activity [24]. They use a wide range of actions to seek to undermine tobacco control, such as through direct lobbying and the use of third parties including front groups, allied industries and academics [25]. However, it is of note that in this analysis most of the reporting suggested that legislative action to prohibit smoking in vehicles carrying children was presented as necessary, enforceable and as having general public support, with the little opposition coming largely from the tobacco industry funded lobby group ‘Forest’. To gain influence in the policy debate, these lobbyists appeared to have focused their arguments around the issue of whether legislation is necessary and how it will infringe smokers’ freedoms, rather than on arguing about the health harms of SHS exposure to children. While the voices opposed to legislation in this study are predominantly those of industry lobby views, Bowditch argues that some social theorists, such as Furedi, also perceive the legislation as a regressive invasion of privacy [26]. Nevertheless, in this media discourse those opposing legislation seemed outnumbered and on the fringe of the central arguments.

Over the decade there was a huge increase in news reports covering this issue, with the greatest occurring after the 2006/2007 smoke-free legislation. This increased volume of coverage is one way in which the news media help propagate and shape public understandings of the harms of SHS to children and potential policy interventions. In a similar study conducted by Freeman et al. [27] examining print media in Australia, over half of the newspaper articles examined used the argument that SHS is harmful to children’s health, a claim only disputed in 4 out of 296 articles. Likewise, our study found few articles arguing these now widely held facts.

Sato [28] has suggested that part of the process of getting issues onto the policy agenda consists of creating a ‘package of ideas’ about the facts and feasible solutions to a problem. Our analysis showed that in presenting the key facts about the problem of SHS to children while in vehicles, articles widely reported on the scale of the problem by presenting information on the number of children exposed to SHS in vehicles. Other well tried tactics, were to question or deny the harmful health effects of products and create controversy about established facts with critics often preferring an educational rather than legislative approach despite it being considered a less effective way of tackling health issues like alcohol and tobacco abuse [29, 30]. Norman et al. [31] further suggest that educational campaigns may have less impact on those in socioeconomically deprived households, who are more likely to be exposed to the effects from public health issues such as SHS.

Some of these facts were identified as key evidence which could be traced back to research studies, including Akhtar and colleagues 2007 survey [3], and more recently Moore et al’s survey conducted in 2012 across 304 primary schools (in Scotland, Wales and Northern Ireland) which showed that post- smoke-free legislation 25.7% (1148 out of 4466) of children “whose family owned a car reported that smoking was allowed in their car” [32]. Similarly to Akhtar et al’s [3] and Moore et al’s [32] results, we found that whilst there may be a growing public awareness of the harms associated with SHS exposure generally, many people were less aware of the particular risks associated with smoking in vehicles carrying children. Some research information was presented throughout the newspaper articles to highlight the issue of children’s developmental vulnerability and susceptibility to the risks of SHS exposure, and the risks posed by the high levels of SHS in confined spaces like vehicles. Considering the former, and consistent with findings reported by the Royal College of Physicians [8], the key facts presented were that children breathe at a faster rate to adults, have a less developed immune systems and are more disposed to various respiratory tract infections.

The work of Semple and colleagues was drawn upon as research evidence throughout the newspaper articles in this study to highlight findings to support the fact that ventilation systems and open windows were insufficient to combat SHS exposure in the vehicle [9, 33]. During car journeys where smoking took place, Semple et al. found that concentrations of fine particulate matter (PM_2.5_) were on average 85 μg/m^3^ three times the World Health Organisation’s 24 hr guidance of 25 μg/m^3^ for indoor air levels. This compared to an average of 7 μg/m^3^ for car journeys where smoking did not occur. As a result, they concluded that children were being exposed to dangerously high levels of SHS in vehicles that allow smoking, even when certain measures are taken to ventilate the air [9]. This suggests that raising public awareness of the wider context of the debate, and in particular, children’s vulnerability to SHS exposure in vehicles could be an area that advocates would do well to continue to address.

In terms of Sato’s suggestion of getting issues onto the policy agenda by offering feasible solutions to a problem, this analysis found a common discourse portraying children as victims of harm from the thoughtless behaviours of adults and thus policy intervention was needed. This presentation is consistent with other studies [34, 35], including Wood et al’s [36] study which analysed the newsprint media portrayal of the ‘harms to others’ from alcohol consumption. Arguments about ‘who’ is harmed and ‘who’ is responsible for smoke-free laws in other countries who have already introduced legislation prohibiting smoking in vehicles carrying children, appear similar. Consistent with Thomson and Wilson [16] and Freeman et al. [27], our analysis suggests that when the focus or concern is on children specifically, opponents against smoke-free laws tend to steer away from criticising this particular aspect of the legislation in their media messages.

Moreover, public opinion surveys which ask about legislation involving the protection of children provoke a great deal of support. Buchanan et al. [37] examined a 2008 YouGov online survey of 3329 adults over the age of 18 living in the UK. This survey reported that 76% of people would support a smoking ban in vehicles carrying children under 18. Similar to our study, arguments against this smoke-free legislation were that it would be unenforceable. This argument has been cited in other studies, perhaps unsurprisingly as critics such as global tobacco companies tend to repeat claims which are translated across different countries [27, 38]. However, it was notable in our analysis and in Freeman et al’s [27] study, that advocates provided examples of laws already enacted successfully in vehicles such as compulsory seatbelt and infant carrier usage, and mobile phone restrictions.

Lobbyist and critics opposing further smoke-free legislation often use an entire host of arguments, remaining consistent across jurisdictions [24]. This was true of the 2006 smoke-free legislation in Scotland which prohibited smoking in enclosed public places. The tobacco industry argued that the legislation would displace smoking to the home, it would cause economic loss, and it would not have any effect on smokers quitting [5]. However, as a by-product of the legislation smoking in the home has decreased [3, 4], smokers say it helped them quit [39], and attitudes towards business and job security have been positive [5]. Nevertheless, messages to undermine such protective legislation are commonly regurgitated in public health debates. Parallels can be drawn in this current study with arguments used to oppose plain packaging for tobacco products [40, 41], minimum unit pricing for alcohol [42] and taxation on fast food products [43], among others.

## Conclusions

In conclusion, the increased level of attention that SHS exposure to children in vehicles is receiving in the print media indicates that this public health issue is gaining in stature. This comes against a backdrop of no mainstream UK or Scottish political party having a manifesto position on this issue during the period of this analysis. Policy advocates might do well to build on this growing debate and to highlight the success of recent smoke-free legislation and of previous legislation relating to in-vehicle and driving behaviour which have promoted a change in social norms. Further, advocacy efforts might target drivers with messages about children’s particular developmental vulnerability to SHS exposure, the dangers posed by smoking in confined spaces such as vehicles, and the appropriate measures that should be taken to reduce the risk of harm.

The role that media coverage of SHS in vehicles has played in formulating debate and reflecting public opinion is likely to have been significant. The recent move towards legislating on a smoking ban in vehicles with children in England by the UK government and the bringing forward of the ‘Smoking (Children in Vehicles) (Scotland) Bill’ by MSP Jim Hume suggests that politicians have caught up with public and scientific opinion on this issue. The harms posed by exposure to SHS in vehicles represent an excellent case-study of the importance of continued media engagement for those involved in developing public health policy.
